# ‘Get in Early’; Biofilm and Wax Moth (*Galleria mellonella*) Models Reveal New Insights into the Therapeutic Potential of *Clostridium difficile* Bacteriophages

**DOI:** 10.3389/fmicb.2016.01383

**Published:** 2016-08-31

**Authors:** Janet Y. Nale, Mahananda Chutia, Philippa Carr, Peter T. Hickenbotham, Martha R. J. Clokie

**Affiliations:** ^1^Department of Infection, Immunity and Inflammation, University of LeicesterLeicester, UK; ^2^Pathology and Microbiology Division, Central Muga Eri Research and Training InstituteAssam, India

**Keywords:** *Clostridium difficile*, *Clostridium difficile* infection, biofilms, *Galleria mellonella*, bacteriophages, bacteriophage therapy

## Abstract

*Clostridium difficile* infection (CDI) is a global health threat associated with high rates of morbidity and mortality. Conventional antibiotic CDI therapy can result in treatment failure and recurrent infection. *C. difficile* produces biofilms which contribute to its virulence and impair antimicrobial activity. Some bacteriophages (phages) can penetrate biofilms and thus could be developed to either replace or supplement antibiotics. Here, we determined the impact of a previously optimized 4-phage cocktail on *C. difficile* ribotype 014/020 biofilms, and additionally as adjunct to vancomycin treatment in *Galleria mellonella* larva CDI model. The phages were applied before or after biofilm establishment *in vitro*, and the impact was analyzed according to turbidity, viability counts and topography as observed using scanning electron and confocal microscopy. The infectivity profiles and efficacies of orally administered phages and/or vancomycin were ascertained by monitoring colonization levels and larval survival rates. Phages prevented biofilm formation, and penetrated established biofilms. A single phage application reduced colonization causing extended longevity in the remedial treatment and prevented disease in the prophylaxis group. Multiple phage doses significantly improved the larval remedial regimen, and this treatment is comparable to vancomycin and the combined treatments. Taken together, our data suggest that the phages significantly reduce *C. difficile* biofilms, and prevent colonization in the *G. mellonella* model when used alone or in combination with vancomycin. The phages appear to be highly promising therapeutics in the targeted eradication of CDI and the use of these models has revealed that prophylactic use could be a propitious therapeutic option.

## Introduction

*Clostridium difficile* is an important human and animal pathogen and a major cause of pseudomembranous colitis where it accounts for 15–39% of antibiotic-stimulated toxin-mediated diarrhea (McFarland, 2009; Viswanathan et al., 2010). *C. difficile* infections (CDI) are becoming increasingly severe due to limited treatment options and the emergence of pathogenic ribotypes (Freeman et al., 2010; Zucca et al., 2013). Complications of the disease can also arise from antibiotic resistance, leading to relapse, increased health care-associated costs and death in 10% cases (Wiegand et al., 2012; Lessa et al., 2015; Vindigni and Surawicz, 2015). Therefore, there is a need for specific and efficient strategies for the targeted eradication of this pathogen.

The pathogenicity of *C. difficile* is linked to potent cytotoxins (toxin A, B and binary toxin AB) which together are responsible for the damage of the epithelial lining leading to pseudomembranous colitis (Lyerly et al., 1988; Carter et al., 2007; Lyras et al., 2009). Other factors such as fimbriae and surface layer proteins contribute to its motility and adhesion to the ileum and caecum where the disease is most prominent (Borriello et al., 1988; Tasteyre et al., 2000). In addition, their hardy spores resist heat and desiccation, thus playing a significant role in their spread, survival and disease (Akerlund et al., 2008; Burns et al., 2011). Of particular relevance to this paper, *C. difficile* also produces biofilms, which consist of aggregates of cells embedded in self-produced extracellular polymetric substance (EPS) (Branda et al., 2005; Dawson et al., 2012; Đapa et al., 2013). The EPS matrix binds the spores and vegetative cells, and provides protection for the bacteria against oxygen stress and enhances their adhesion to abiotic surfaces (Dawson et al., 2012). Therefore, biofilms contribute to *C. difficile* virulence by potentially enhancing persistence and proliferation of the pathogen in the environment, and during active infection where they could interfere with the activity of antimicrobial agents and treatment of the disease.

The conventional CDI treatment relies solely on three antibiotics: metronidazole, vancomycin and fidaximicin, although limitations to their use have been reported (Aslam et al., 2005; Pepin, 2008; Crawford et al., 2012). Metronidazole is not effective for the treatment of all ribotypes, vancomycin is predominantly used as a treatment of last resort due to the possibility of resistance emergence. Fidaximicin is much newer and is not cost effective as a first-line treatment for some strain-specific CDIs (Wiegand et al., 2012; Bartsch et al., 2013; Chilton et al., 2014). In terms of mechanisms of action, metronidazole disrupts DNA replication, vancomycin targets the bacterial cell wall and fidaximicin hinders RNA polymerase activity (Watanakunakorn, 1984; Löfmark et al., 2010; Venugopal and Johnson, 2012). Clearly antibiotic action is hampered by the limited access to the *C. difficile* due to obstacles posed by the hardy spores and biofilm formation. Pertinent to the biofilm is the EPS matrix which could be overcome by antimicrobial agents such as bacteriophages (phages) (Parasion et al., 2014; Chan and Abedon, 2015; Abedon, 2016).

Phages are viruses that specifically infect bacteria. By infecting and then lysing biofilm-causing bacteria they have been shown to prevent biofilm formation (Parasion et al., 2014; Chan and Abedon, 2015; Dalmasso et al., 2015; Abedon, 2016). In addition, the EPS-depolymerases carried by some phages, which have a different function than lysins (lysis of the bacterial cell), impact biofilms by degrading EPS matrix (Parasion et al., 2014; Chan and Abedon, 2015). This enzyme activity presumably exposes the bacterial cells to antimicrobial agents, and provides access for phages to the receptors found on the cell wall, leading to infection and lysis. These advantages of phages over antibiotics is attractive and could be exploited either for phage mediated eradication of *C. difficile*, or to enhance antibiotic activity.

Phages have been developed for therapeutic purposes to treat bacterial infections caused by *Staphylococcus*, *Streptococcus*, *Pseudomonas*, *Proteus*, and *Escherichia coli* (Slopek et al., 1987; Abedon et al., 2011; Brüssow, 2012). In addition to medical uses, their safety, specificity and ability to replicate *in situ* has meant that phages both can and are currently used in the food industry as alternatives to antibiotics and as decontamination agents (EBI Food Safety, 2007; Hagens and Loessner, 2010; Kumari et al., 2011). Although several *C. difficile* phages have been characterized, few studies have focused on their potential applications for the treatment of CDI (Zucca et al., 2013; Hargreaves and Clokie, 2014; Nale et al., 2016). Most therapeutic studies have investigated single phages, and reported the isolation of resistant and lysogenic clones (Ramesh et al., 1999; Govind et al., 2009; Meader et al., 2010, 2013; Nale et al., 2016). In contrast, our previous work demonstrated that the use of optimized phage combinations could mitigate lysogeny and resistance *in vitro*, and could reduce colonization and extend the life expectancy of animals in a hamster model of CDI (Nale et al., 2016).

The other previous work on therapeutic CDI phages has concentrated on exploiting an artificial human gut model (Meader et al., 2010, 2013), and on the hamster model (Ramesh et al., 1999; Govind et al., 2011; Nale et al., 2016). Both are useful but have limitations. The artificial human gut model has been used to reveal many facets of enteric pathogens but is has a large footprint and the experiments are technically difficult to run. Although the hamster CDI model is good due to the ability of the animals to demonstrate various clinical symptoms of the disease that are comparable to humans, these animals are susceptible to the toxins, and succumb to the disease easily (Price et al., 1979; Best et al., 2012). The technical limitations of these models, the lengthy process for ethical/licenses approvals, and limitations associated with cost and space are causing researchers to shift interest toward exploring other substitutes for *C. difficile* research. Recent studies, for example, have examined zebra fish embryos to test *C. difficile* toxin B clones (Hamm et al., 2006; Lanis et al., 2010).

*Galleria mellonella* larvae have been reported to be a suitable alternative model to larger mammals for bacterial colonization studies (Ramarao et al., 2012) and an excellent tool for pharmacokinetics studies of antimicrobials (Hill et al., 2014). The model has also been used to study pathogenesis of bacteria such as *Pseudomonas aeruginosa*, *Listeria monocytogenes, Bacillus cereus*, and *Francisella tularensis* (Miyata et al., 2003; Aperis et al., 2007; Fedhila et al., 2010; Mukherjee et al., 2010) and phage therapy for *Burkholderia cepacia* and *P. aeruginosa* (Seed and Dennis, 2009; Beeton et al., 2015). However, no studies have been published to date to examine how useful *Galleria* would be as a model for CDI colonization, or subsequent phage therapeutic studies. There have been limited studies on the optimal dosing of phages and it is likely that significant amounts of knowledge can be gained from this model to inform subsequent animal experiments.

In this study, we examined the ability of *C. difficile* phages to target this bacterium in two models of infection. We investigate the efficacy of an optimized phage cocktail to prevent or treat *C. difficile* biofilms, and as a stand-alone treatment or combined with vancomycin in the *G. mellonella* larvae CDI model. We show that the phages prevent biofilm formation *in vitro*, and penetrate into and reduce established biofilms. In the *Galleria* model, the phages are most effective when used prophylactically, and in the presence of vancomycin, with multiple doses required to produce comparable results in the remedial regimen. It is clear that phages are able to impact *C. difficile* biofilms and significantly reduce the extent of colonization in a *G. mellonella* model of CDI and therefore appear to have great potential for the treatment of this bacterial pathogen. These models can be used to pursue aspects of phage therapy development in the future, such as to develop dosing and timing regimens.

## Materials and Methods

### *In vitro* Phage Treatment of Biofilms

#### Bacterial Isolates and Phages

Six *C. difficile* ribotype 014/020 human clinical strains (CD105LC2, ATJ, AIP, ATK, TL176 and AUS1022) were examined in this study (Nale et al., 2016). The biofilm characteristics of R20291 were previously established and therefore was used as a control strain in the biofilm preliminary assays (Dawson et al., 2012; Đapa et al., 2013). The four phages examined, CDHM1, 2, 5, and 6 were previously isolated from our laboratory and propagated individually on the environmental *C. difficile* strain, CD105HE1 (Hargreaves et al., 2014; Nale et al., 2016). The filtered phage lysates (using 0.22 μl filters, Merck Millipore Ltd, Cork, Ireland) were mixed equally to form the cocktail. All the bacterial strains and phages tested were preserved in 25% glycerol stocks at -80°C.

#### Establishment of Biofilm Characteristics of the Bacterial Strains

Prior to phage treatment, the biofilm characteristics of the test strain CD105LC2 was first established *in vitro* on 12-well plates (Greiner Bio-One Ltd, UK) and the feature compared with the other five ribotype 014/020 strains using methods previously described (Đapa et al., 2013). Briefly, 48 h *C. difficile* cultures were produced on brain heart infusion (BHI) (Oxoid, UK) agar plates supplemented with 7% defibrinated horse blood (TCS Biosciences Ltd, UK). Broth cultures were prepared by inoculating a bacterial colony into 5 ml brain heart infusion supplemented (BHIS) with 0.1% w/v each of Ly-cysteine (Sigma–Aldrich, UK) and 5 mg/ml yeast extract (Oxoid, UK) and incubated at 37°C anaerobically for 18–24 h. Afterward, 1:10 dilutions of the overnight cultures (10^7^ CFU/ml) were prepared in pre-reduced BHIS broth and 3 ml aliquots were added to the 12-well plates and incubated for 1–5 days. After each day the planktonic and biofilm vegetative cells and heat-resistant (at 65°C for 3 h) spores were enumerated on BHI agar and Brazier’s cefoxitin, cycloserine and egg yolk (CCEY) agar plates (BioConnections, UK) respectively. To determine the biofilm mass, planktonic cultures from the wells were carefully removed to expose the thin biofilm layer at the bottom. The wells were washed twice with sterile phosphate buffered saline (PBS) and stained with 2 ml 0.1% filtered crystal violet followed by incubation at room temperature for 1 h. After washing off the crystal violet three times with PBS, 1 ml of absolute methanol was applied and incubated at room temperature for 5 min to extract the biofilm-bound stain. Approximately 250 μl was transferred to a 96-well plate and absorbance was read at 595 nm (Dawson et al., 2012; Đapa et al., 2013).

#### Phage Treatment of Biofilms

Having established the biofilm characteristics of the test strain, CD105LC2, a fresh biofilm of this strain was produced from a dilution of the overnight culture (Set 1) and subjected to four different phage treatment regimens (Sets 2–5, **Table 1**) using 300 μl of the single or phage combination (at 10^9^ PFU/ml, MOI = 10). Sets 2, 3, and 4 represent biofilm pre-treatment regimens where phages were added: 1 h prior to bacterial exposure (Set 2), 1 h post-bacterial inoculation (Set 3) or simultaneously with the bacteria (Set 4). The only post- treatment regimen was Set 5. In this treatment, maximum biofilms (at 24 h) were established first and planktonic cultures removed as described above before treating them with the phages as shown in **Table 1**. The treated biofilms were further incubated for 24 h. After each treatment, the resultant vegetative cells and spores were enumerated and the biofilm mass determined as described above.

**Table 1:** Experimental set-up for the pre- and post-biofilm regimens with single and combined phages.

Regimen	Biofilm phage treatment regimens	Treatment
Set 1	Control (Untreated biofilms)	3 ml of 10% dilution of ON bacterial culture in pre-reduced BHIS was added to the wells.
Set 2	Pre-treatment 1	3 ml of the 10% diluted culture was added to the wells and incubated for 1 h. Afterward, 300 μl phage lysate/cocktail was added.
Set 3	Pre-treatment 2	300 μl of phage lysate/cocktail was added to the wells and incubated for 1 h. After incubation, 3 ml of 10 % diluted culture was added.
Set 4	Pre-treatment 3	3 ml of 10% diluted bacterial culture was mixed with 300 μl of phages and added to the wells.
Set 5	Post-treatment	3 ml of 10 % diluted bacterial culture was added to the plates and incubated overnight. Afterward, the planktonic cells were washed out and 300 μl of phage lysate/cocktail was added.

*Phages were propagated individually (10^9^PFU) and mixed together in equal proportions to constitute the cocktail. Biofilm treatment was conducted with 300 μl of the phage cocktail and 3 ml of the 10% diluted overnight bacterial cultures (MOI = 10) or on established biofilms of a C. difficile 014/020 isolate (CD105LC2). All treated biofilms were further incubated for 24 h before enumeration of the vegetative cells and spores.*

#### Phage Treatment of Colony Biofilms

To determine the effect of phage treatment on the topology of biofilms, colony biofilms were produced on membrane filter disks (Merck Millipore Ltd, Ireland) and treated with the phage cocktail. To do this, the filter disks were sterilized both sides using Stratalinker UV crosslinker 2400 (Strategene, US) at 9999 μm setting. The sterilized disks were aseptically transferred onto BHI agar plates and 5 μl of a 10-fold dilution of the overnight bacterial culture was applied onto the filter disks. After 24 h incubation, a set of membrane disks for each day was transferred onto a fresh BHI agar plate and this was repeated up to 5 days. For phage treatments, 50 μl of the phage cocktail was gently applied to cover the entire surface of the biofilm after transferring biofilms to fresh medium each day. The treated biofilms were further incubated for an additional 24 h. Biofilms for each day (phage-treated and control) were removed and transferred to a tube containing 1 ml of cold BHI and vortexed (3000 rpm/min for 30 s) to dislodge the biofilm from the membranes. The membranes were removed and the resultant cultures were washed three times in ice-cold BHI and centrifuging at 15 000 *g* for 5. The vegetative cells and spores were enumerated using methods described above.

#### Scanning Electron and Confocal Microscopy

To conduct scanning electron microscopy (SEM) analysis on the biofilms, a set of phage-treated and control biofilms on the membrane disks were transferred to 12-well plates and sterilized with 1 ml of 2.5% gluteraldehyde (v/v in PBS) for at least 24 h. The membranes were processed through several washes in distilled, de-ionized water, followed by 30-min dehydration steps through a 30, 50, 70, 90, and 100% ethanol series. After two more 20-min washes through 100% analytical grade ethanol, the membranes were gradually infiltrated with Hexamethyldisilazane (HMDS) by 1 h washes in 2:1, 1:1 and finally 1:2 mixtures of ethanol:HMDS, followed by two 30-min washes in 100% HMDS. The HMDS was removed, and membranes were air dried overnight. Dried membranes were mounted onto 12.5 mm aluminum stubs and sputter coated with gold/palladium using a Quorum Q150T ES coating unit. Samples were observed, and the images recorded using Hitachi S3000H scanning electron microscope with an accelerating voltage of 10 kV.

For the confocal analysis, a different set of phage-treated and untreated colony biofilms were prepared on membrane filters as described and stained using FilmTracer Live/Dead biofilm Viability kit according to the manufacturer’s recommendations (ThermoFisher Scientific, UK). Membranes were sterilized with the glutaraldehyde for 24 h. Confocal fluorescence imaging was conducted as previously described (Osman et al., 2016).

### *In vivo* Analysis of Phage Therapy Using the *Galleria mellonella* Model

#### Preparation of Bacterial Inoculum

A dilution of the overnight inoculum of the bacteria was prepared as above and incubated until OD_550_ of 0.2 was attained. The culture was then centrifuged at 15 000 *g* for 5 min and the resultant pellet was re-suspended in cold BHI to a final concentration of 1 × 10^7^ CFU/mL.

#### Bacterial Infection, Colonization and Treatment of *G. mellonella* Using a Single Phage Dose

Larvae of *G. mellonella* were obtained from Live Food UK Ltd. (Rooks Bridge, UK). On arrival, the larvae were stored immediately at 4°C and used within 1 week. Larvae with approximate weight of 0.25–0.30 g were selected for *in vivo* analysis (Abbasifar et al., 2014). The larvae were surface-sterilized with cotton swaps dipped in 70% ethanol. A single dose (in 10 μl) of either 10^5^ CFU of bacterial inoculum, 10^6^ PFU of phage cocktail or BHI broth was administered via the oral route using a 10-μl Hamilton syringe pump. Four randomly selected larvae were examined in each treatment regimen at 37°C in plastic Petri dishes. The insects remained unfed throughout the experiment (Ramarao et al., 2012). Two control groups were observed: the first received only bacteria (Control CD105LC2) and the second a dose of the phage cocktail (Control Phage). The treatment models were divided into either prophylactic or remedial groups. In the prophylactic model, phages were administered 2 h before bacterial infection (Phages + CD105LC2) or given simultaneously with the bacteria (Phages + CD105LC2 (s)). In the remedial group, bacteria were administered 2 h before the phage treatment (CD105LC2 + Phages).

Insects were considered dead when they become inert and turned black in color (Ramarao et al., 2012). Experiments were repeated three times. Survival curves were plotted using the Kaplan-Meier method in GraphPad Prism 6, and differences in survival rates were calculated by using the Log-rank (Mantel-Cox) test.

#### Bacterial and Phage Recovery from the Larvae

At the end of the experiment, the larvae were dissected dorsoventrally, the heamolymph extracted into ice-cold BHI, and phage and bacteria enumerated as described above. *C. difficile* colonies were confirmed with colony PCR targeting the *C. difficile* 16S rRNA (Rinttilä et al., 2004). For recovery of phages, homogenized heamolymph mixtures were centrifuged at 5000 *g* for 10 min at 4°C and the filtered supernatants were assayed for phages using spot test on the phage propagating host, CD105HE1, as the indicator strain. Recovery of phages from the feces was conducted by combining and suspending excreta from all four larvae in a group into 1 ml of cold BHI and incubated at 4°C for 1 h. The centrifuged and filtered supernatants were enumerated for phage presence as above. Data were analyzed using GraphPad Prism 6. All *in vivo* experiments were performed at least thrice.

#### *G. mellonella* DNA Extraction and qPCR

Heamolymph from the four larvae from the treatment groups above were combined and DNA was extracted from them using DNeasy Blood & Tissue Kit (Qiagen, Germany). Approximately, 50 ng of DNA from each group of larvae was subjected to RT-PCR targeting the *C. difficile* 16S rRNA using 7500 Fast Real Time PCR system with Fast SYBR Green Master mix (Thermo Fisher Scientific, USA). Data was compared with standard CFU/ml counts in the growth curve of the test strain and analyzed using GraphPad Prism 6.

#### Treatment of *G. mellonella* Using Multiple Phage Doses

Since the above phage treatment was conducted using a single dose of the phage cocktail, further experiments were conducted to determine if multiple phage doses could improve the treatment regimen. To do this, five groups of larvae were given a mixture of phages/bacteria at the 0 h. This is the only treatment in group A, but in group B larvae subsequently received an additional single dose of phage cocktail at 6 h while group C received 2 doses at 6 and 12 h (**Table 2**). Group D larvae received three doses of phages at 6, 12 and 24 h while group E received 4 doses at 6, 12, 24, and 48 h (**Table 2**). Survival rates were recorded every 12 h for 72 h.

**Table 2:** Multiple phage dose regimens observed in the remedial treatments on *G. mellonella* larvae model of CDI.

Time to phage dose (h)	Phage dose regimens					
	Control	A	B	C	D	E
0	B	P, B	P, B	P, B	P, B	P, B
2			P	P	P	P
6				P	P	P
24					P	P
48						P
72	Cull	Cull	Cull	Cull	Cull	Cull

*Larvae were inoculated with a mixture of phages (P) (10^6^PFU) and/or bacteria (B) (10^5^CFU) in 10 μl volume and subsequently treated with additional doses of phages at the indicated time points. At the end of the experiment larvae were killed (culled) by placing them in -20°C for 5 min.*

#### Phage/Antibiotic Combined Treatment on the Larvae

After phage therapy was established on the larvae, the phages were tested as adjunct to vancomycin, a commonly used antibiotic for CDI. Prior to the combined treatment, the windows of opportunity during which phages may be most effective as prophylactic therapeutics was determined by administering the phages to the larvae first. Time delays of 2 h (A, Phage prophylactic 1), 4 h (B, Phage prophylactic 2), 6 h (C, Phage prophylactic 3) or 12 h (D, Phage prophylactic 4) were observed before exposing them to bacterial infection (**Table 3**). To determine if the phage prophylaxis would aid vancomycin treatment, insects were given phages and in 2 h intervals challenged with bacteria and given vancomycin (64 μg/g, MIC ≥ 1 μg/ml) (E, Phage prophylaxis 5). Similarly, the ability of vancomycin prophylaxis to enhance phage therapy was also tested in treatment F (vancomycin prophylaxis) where vancomycin was given first and subsequently bacteria and phages were given at 2 h intervals respectively (Desbois and Coote, 2011). The treatments were compared with vancomycin treatment, (G, vancomycin given 2 h after exposure to bacteria) or vancomycin/phage pre-treatments, (H, vancomycin and phages were given simultaneously before exposure to bacteria 2 h later) or phage/vancomycin remedial regimen (I, vancomycin/phage simultaneously given 2 h post bacterial challenge) (**Table 3**).

**Table 3:** Phage prophylaxis regimens and phage/vancomycin combined treatment in *G. mellonella* larvae.

Time (h) →	0	2	4	6	12	24	25	60	72
Treatments↓
A, Phage prophylaxis 1	P	B						Cull	
B, Phage prophylaxis 2	P			B				Cull	
C, Phage prophylaxis 3	P				B			Cull	
D, Phage prophylaxis 4	P					B		Cull	
E, Phage prophylaxis 5	P	B	V					Cull	
F, Vancomycin prophylaxis 1	V	B	P					Cull	
G, Vancomycin treatment	B	V						Cull	
H, Vancomycin/phage prophylaxis	VP	B						Cull	
I, Vancomycin/phage remedial regimen	B	VP						Cull	
J, Clindamycin pre-treatment 1	C					B	P		Cull
K, Clindamycin pre-treatment 2	C					P	B		Cull
L, Control bacteria	B							Cull	

*The larvae were inoculated with 10 μl of phages (P), vancomycin (V) and/or clindamycin (C) at the indicated times. Bacterial enumeration from each larva was conducted from inactivated (culled) larva.*

Because CDI takes advantage of dysbiosis in the gut, another group of larvae were first treated with clindamycin (30 μg/g, MIC ≥ 1.125 μg/ml) to suppress bacterial commensals in the haemolymph. Subsequently, after 24 h the larvae were exposed to either bacteria followed by a dose of phage cocktail (J, Clindamycin pre-treatment 1) or given phage first before the bacteria (K, Clindamycin pre-treatment 2). All treatment groups were compared to the control bacterial group (L, which received just bacteria) (**Table 3**).

Larvae were culled at the 60th hour except for groups J and K, which required additional 12 h after the last exposure (**Table 3**).

## Results

### *C. difficile* Strains Have Different Biofilm Characteristics

To determine the characteristics of the test strain, CD105LC2, prior to phage treatment, we first established its ability to produce biofilms and compared this feature to other ribotype 014/020 strains and to the reference strain, R20291 (ribotype 027). In preliminary biofilm assays, absorbance readings showed that maximum biofilm was produced at 24 h. Therefore, all subsequent analyses were conducted on biofilms formed at this time. The ribotype 014/020 strains produced different levels of biofilm (Supplementary Figure S1A). ATJ, AIP, AUS1022 and TL176 were similar at OD_595_ ~1.1, while the other two strains, ATK and CD105LC2 had readings of ~1. 5. The biofilm produced by R20291 strain (OD_595_~1.7) at 24 h is comparable to previously published data (Dawson et al., 2012; Đapa et al., 2013). Analysis of viability counts also suggests that variable quantities of spores/vegetative cells are produced by the ribotype 014/020 strains both in the biofilms as well as in the planktonic cultures (Supplementary Figure S1B). Generally, there were higher spore counts in the planktonic cultures (10^5^–10^7^ CFU/ml) than in the biofilms (10^2^–10^4^ CFU/ml) but higher levels of vegetative cells in the biofilms (10^6^–10^8^ CFU/ml) compared to the planktonic cultures (10^2^–10^4^ CFU/ml) (Supplementary Figure S1B).

### Phage Treatment Regimens on *C. difficile* Biofilms

**FIGURE 1:**
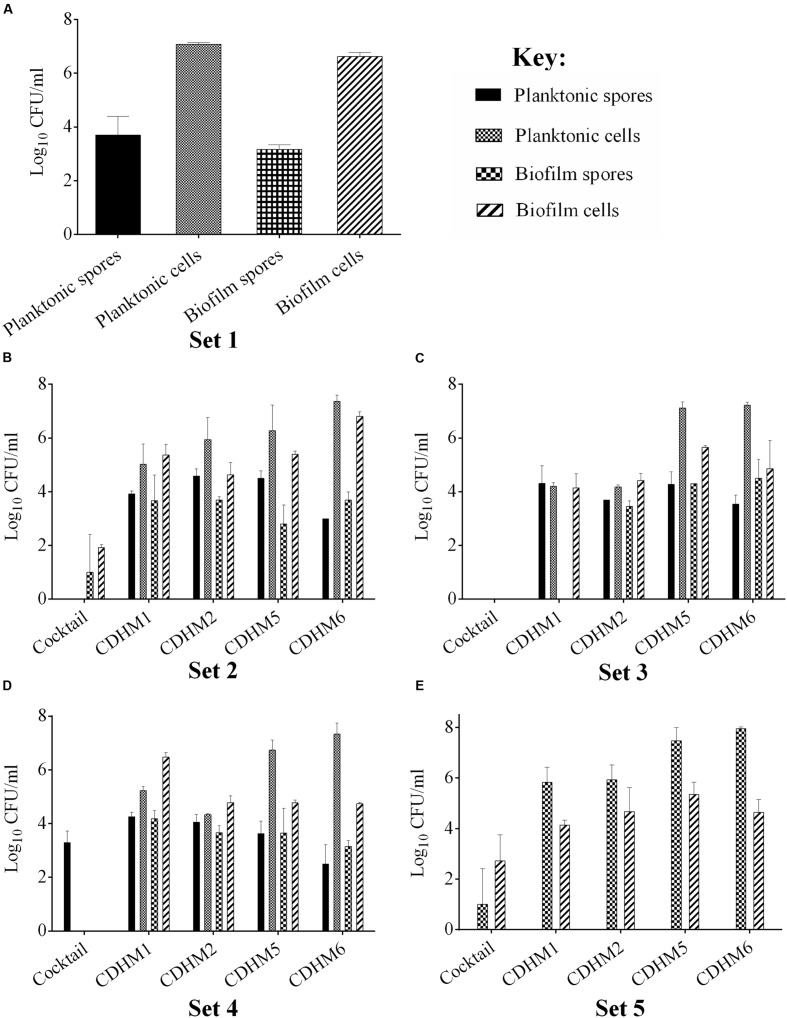
**Bacterial recovery from biofilms in pre- and post-biofilm treatments with combined and individual phages (CDHM1, 2, 5 and 6). (A)** Set 1 represents the untreated biofilms while Sets 2–4 represent the pre-treatment regimens, where the phages were added after bacterial growth for 1 h (Set 2, **B**), phages were incubated for 1 h before bacteria (Set 3, **C**) or mixed together before adding to plates (Set 4, **D**). **(E)** Represents viability counts after phage treatment post-biofilm formation. Experiments were conducted with three replicates and repeated twice. Data was analyzed using GraphPad Prism 6. Error bars are SEM of all replicates.

Having established the biofilm characteristics of the test strain, we determined the impact of phages on biofilm formation. We examined the activity of the individual and combined phages when applied before or after the biofilm establishment, compared to the untreated biofilm (Set 1, **Figure 1A**). In all of the pre-treatment regimens examined (Sets 2–4), vegetative cells were completely eliminated in the planktonic cultures when treated with the phage combination (**Figures 1B–E**). When bacterial growth was established for an hour before phage treatment (Set 2), vegetative cells were eliminated but ~10 and ~ 10^2^ CFU/ml of spores and vegetative cells, respectively, were detected in the biofilms (**Figure 1B**). Interestingly, when the phages were added first (Set 3), biofilm establishment was prevented and no bacteria were detected either in the biofilm or planktonic cultures (**Figure 1C**). When bacteria and phages were added simultaneously (Set 4), ~10^3^ CFU/ml of spores were recorded in the planktonic culture (**Figure 1D**). In the post-biofilm regimen (Set 5), phages were less effective and ~10^2^ and 10^4^ CFU/ml vegetative cells and spores were recovered respectively (**Figure 1E**). In contrast to treatments with the phage combination, the single phage treatments were less effective (**Figures 1B–E**).

### Bacterial Enumeration and Microscopy Analysis Confirmed That Phages Can Penetrate *C. difficile* Biofilms

**FIGURE 2:**
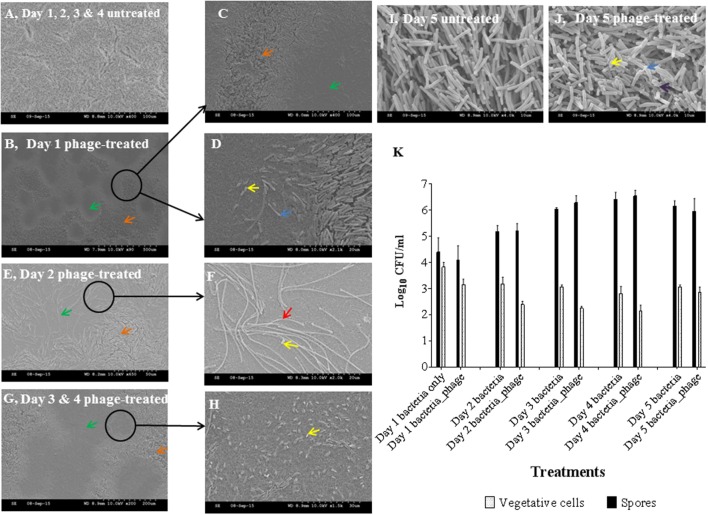
**Scanning electron microscopy (SEM) analyses and bacterial enumeration from established *C. difficile* (CD105LC2) colony biofilms treated with phage combination. (A)** Topology of untreated biofilm. Impact of phage cocktail on topologies of Day 1 **(B–D)**, Day 2 **(E,F)**, Day 3 **(G)** and 4 **(H)** and Day 5 (untreated **I** and treated, **J**) biofilms are shown. Green arrows, plaques (lysed zones); orange arrows, unaffected areas; blue arrows, spore mother cells; yellow arrows, spores; red arrow, elongated cells, and black arrows, disintegrated cells. Graphical representation of viability counts for spores and vegetative cells are shown in **(K)**.

To further investigate the effect of phages on the established biofilms, we used scanning electron and confocal microscopy to determine the physical changes in the properties of the colony biofilms. The topology of the untreated days 1, 2, 3, and 4 biofilms have evenly distributed cells (**Figure 2A**). In contrast, the surface of the phage-treated day 1 biofilms had distinct lysed zones (plaques, represented by green arrows) of ~180–300 μm diameter distributed on the surface but intact bacterial cells were observed around the edges of the lysed zones (orange arrows) (**Figures 2B,C**). Under higher magnifications, it was clear that phage lysis was selective for vegetative cells only, leaving the spores (yellow arrows) and spore mother cells intact (blue arrows) (**Figure 2D**). Examination of the lysed zone of day 2 biofilms (**Figures 2E,F**) revealed elongated cells measuring 15–100 μm in length (indicated using a red arrow) and spores (**Figure 2F**). These elongated cells were only present in the lysed parts of the biofilm. Similar lysed zones were seen in the phage treated biofilms after 3 and 4 days (**Figures 2G,H** respectively) but more spores were seen in day 4 biofilm (**Figure 2H**). At day 5, no plaques were observed but spores, spore mother cells and disintegrated cells (indicated using a purple arrow) were observed in the control and phage-treated biofilms (**Figures 2I,J** respectively).

Phage treatment on the biofilm resulted in a ~0.5–1 log reduction in vegetative cells for 1–4 days old biofilms, but a negligible effect on the day 5 biofilm cells was observed. Spore formation also progressed daily despite phage treatment (**Figure 2K**).

**FIGURE 3:**
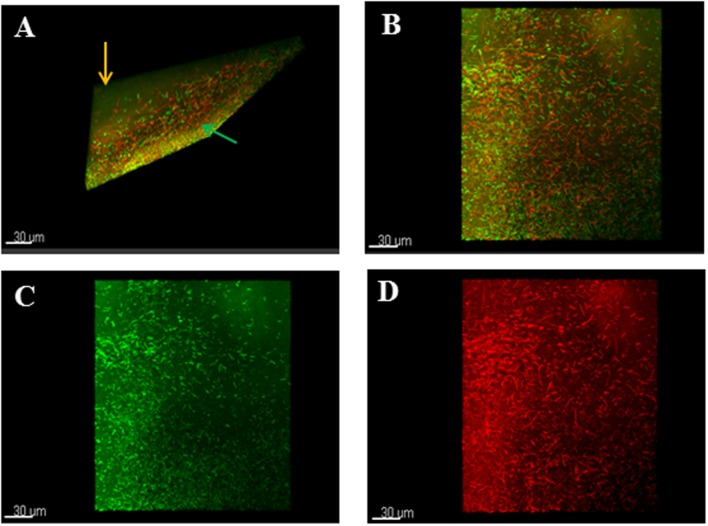
**Confocal microscopy analyses of stained phage-treated colony biofilms. (A)** T-section through a 24 h colony biofilm treated with phages and stained with Live/Dead viability staining kit showed lysed zones (green arrow) and unaffected areas (orange arrow). **(B)** Shows mixture of live/dead cells in the biofilm. **(C)** Shows normal bacterial morphology of the live cells. **(D)** Shows elongated dead cells in the biofilm. Biofilms were treated with Live/Dead Viability Kit, which stained live cells green and the dead cells red by the Syto 9 and propidium iodide dyes respectively.

Analysis of live or dead bacteria in the biofilms using differential staining and confocal microscopy confirmed the lysed zones (green arrows) and the unaffected areas around the zones (orange arrows) (**Figure 3A**). In addition, the elongated cells observed in the lysed zones were dead cells as stained by the red propidium iodide dye (**Figures 3B–D**).

### Efficacy of Phage Prophylactic and Remedial Regimens on *G. mellonella* Larva

To further test the ability of the phages for prophylactic or remedial treatment for CDI we determined their efficacy in the *G. mellonella* larvae using the same bacterial strain and phage combination used for the biofilm assays.

Prior to the bacteria infection, we confirmed that the BHI medium used to suspend the phages and bacteria had no significant effect on the larvae (Supplementary Figure S2). The stability of the phages within the larvae was determined by administering the phages via the oral cavity and analyzing phage recovery from the feces and gut of the sacrificed insects using spot test. Phages remained stable and recoverable from the insects for up to 5 days (Supplementary Figure S3). To determine the pattern of CD105LC2 colonization within the insects, a range of doses were tested and we showed that 10^5^CFU caused the death of all insects within 60 h, with first fatal case recorded in the first 24 h post-infection (Supplementary Figure S2). As expected, colonization progressed with time and approximately 10^8^ CFU/larva of bacteria load was recovered at the end of the treatment as opposed to ~10^4^ CFU/larva starting bacterial load (Supplementary Figure S4).

**FIGURE 4:**
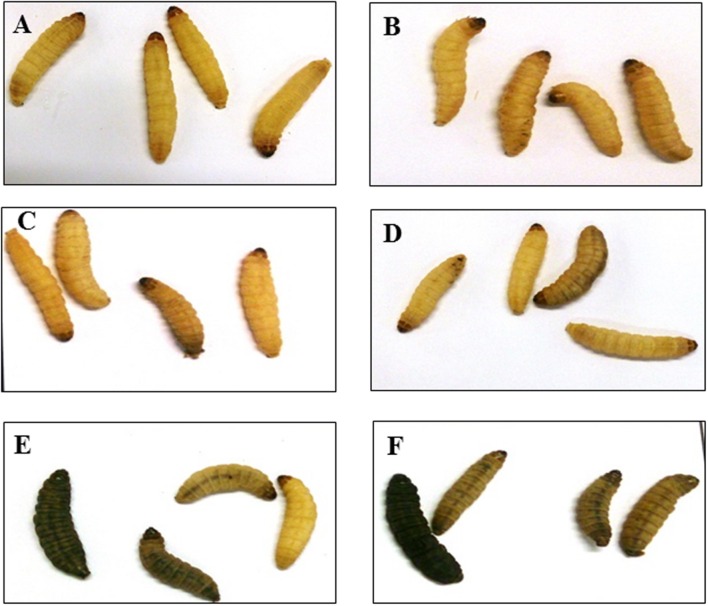
**Impact of phage treatment on the morphology and survival of *G. mellonella* larvae at the end of 60 h.** Larvae were treated with either 10^5^ CFU of bacteria and/or 10^6^ PFU of phages. The untreated **(A)**, phage-only inoculated larvae **(B)** and prophylaxis group **(C)** remained healthy and yellow in color at the end of the experiment. While some of the larvae in groups which received a mixture of phages and bacteria **(D)** and the remedial group **(E)** turned black in color. All larvae in the control bacterial group **(F)** turned black in color at the end of the treatment. Larvae are considered dead when they turn black and remain motionless.

**FIGURE 5:**
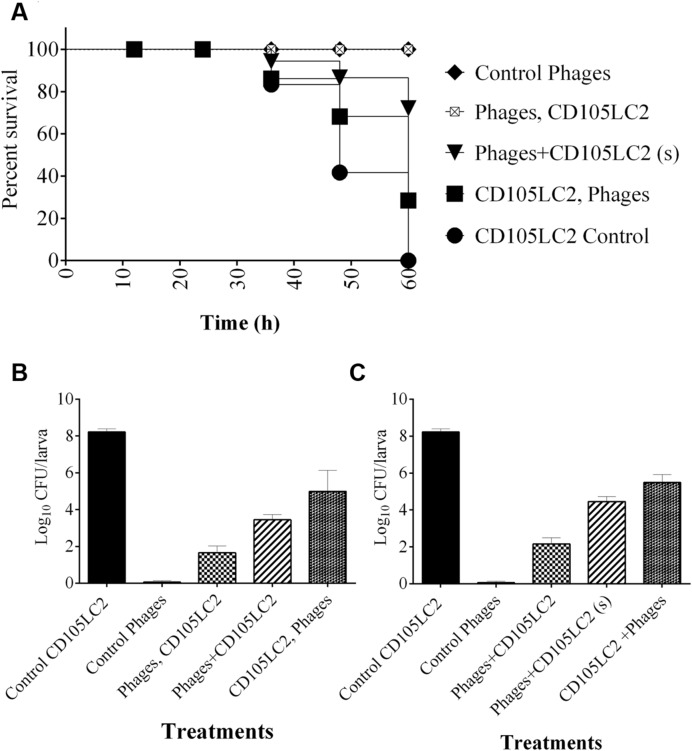
**Impact of phage treatment on *C. difficile* colonization and survival rates of *G. mellonella* larvae CDI model. (A)** Survival curve showing impact of phage treatment on the larvae in the prophylaxis group (phages, CD105LC2), remedial (CD105LC2, Phages) regimens or larvae treated with a mixture of phages and bacteria simultaneously (phage + CD105LC2 (s)). The treatments were compared with control groups where larvae were given phages only (Control phages) and those that received bacteria only (CD105LC2 control). Bacterial enumeration on CCEY plates are shown in **(B)**, while **(C)** represents qPCR absolute quantification data targeting *C. difficile* 16S rRNA and comparing with standard CD105LC2 curve. A statistical significant difference in time to death was observed (*p* < 0.0001) using four larvae per group and experiment was repeated three times. Error bars are SEM of all the replicates. Data was analyzed using GraphPad Prism 6.

To determine phage efficacy in this model, 3 regimens were conducted on the insects. Survival and bacterial load were ascertained and compared to untreated group (**Figure 4A**) and to larvae inoculated with phages only (**Figure 4B**). Where the insects were treated with phages 2 h before inoculating them with the bacteria, the insects were completely protected with 100% survival rate within this group (**Figures 4C** and **5A**). This is comparable to the phage control group (**Figure 4B**). When the larvae were given both the phages and the bacteria simultaneously, efficacy was reduced to 98, 85, and 72% at the 36, 48, and 60 h respectively post inoculation (**Figures 4D** and **5A**). The least effective treatment was recorded in the remedial group where phages were added 2 h post-bacteria. Infection resulted to 82, 65, and 30% survival at the three time points (**Figures 4E** and **5A**). All larvae were dead at the end of the experiment, however, (**Figures 4F** and **5A**). The bacterial numbers at the end of the experiments were consistent with results from the survival assays (**Figures 5B,C**). The prophylactic group (Phages, CD105LC2) had the lowest bacterial load with ~10^2^ CFU/larva recovered while ~10^6^ CFU/larva were observed with the remedial group and ~10^4^ CFU/larva when phages/bacteria were administered simultaneously (**Figure 5B**). The treatments were statistically significantly different (*P* < 0.0001). A similar trend was observed with the qPCR data (**Figure 5C**). Sixty isolates were recovered from all the treatment and testing them with the phage combination showed that the isolates were still sensitive to the phages.

### Effect of Multiple Phage Doses on *C. difficile* Colonization and Survival in the *Galleria* Model

**FIGURE 6:**
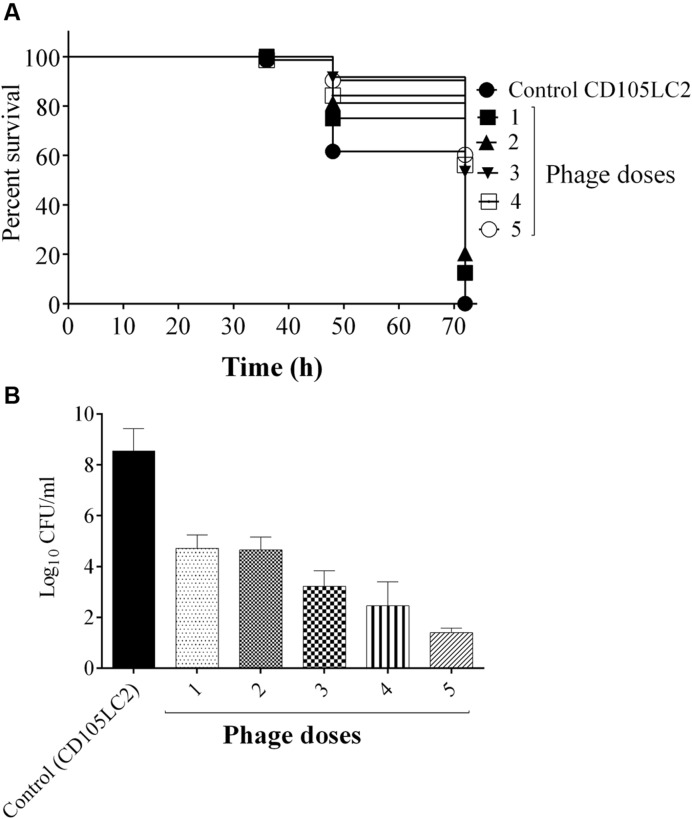
**Impact of multiple phage doses on *G. mellonella* larvae CDI model.** Larvae were treated with a mixture of bacteria and phages at the beginning of the experiment (one dose). Subsequently, the larvae received single increment phage doses at 2, 6, 12, and 24 h, which represent 2, 3, 4, and 5 doses respectively. Impact of phage treatment on larvae which received all the dose regimens at the time points are presented. **(A)** Survival rates of larva treated with the different doses. **(B)** Bacterial enumeration from the larvae at the end of the experiment. Four larvae were examined in each group and experiment was repeated three times. A statistical significant difference in time to death was observed (*p* < 0.0001). Error bars represent SEM of all replicates. Data was analyzed using GraphPad Prism 6.

In an attempt to improve the efficacy of the phages administered post-infection, we determined if the use of multiple phage doses could improve the remedial treatment. This was indeed the case and larvae given a single dose and 2 doses had the lowest survival rates of ~10 and 20% respectively, while groups that received 3, 4, or 5 doses showed an increasing improvement with survival rate of ~60% at the end of the experiment (**Figure 6A**). Treatments differences here were also statistically significant (*P* < 0.0001). Again bacterial recovery from the larva was consistent with decreased bacterial numbers as the number of phage dosages increased. Approximately, 10^5^ CFU/larva were recovered from the single dose group as opposed to 10^2^ CFU/larva from the group, which received five doses (**Figure 6B**).

### Phage Prophylactic Regimens and Phage/Antibiotic Treatments

**FIGURE 7:**
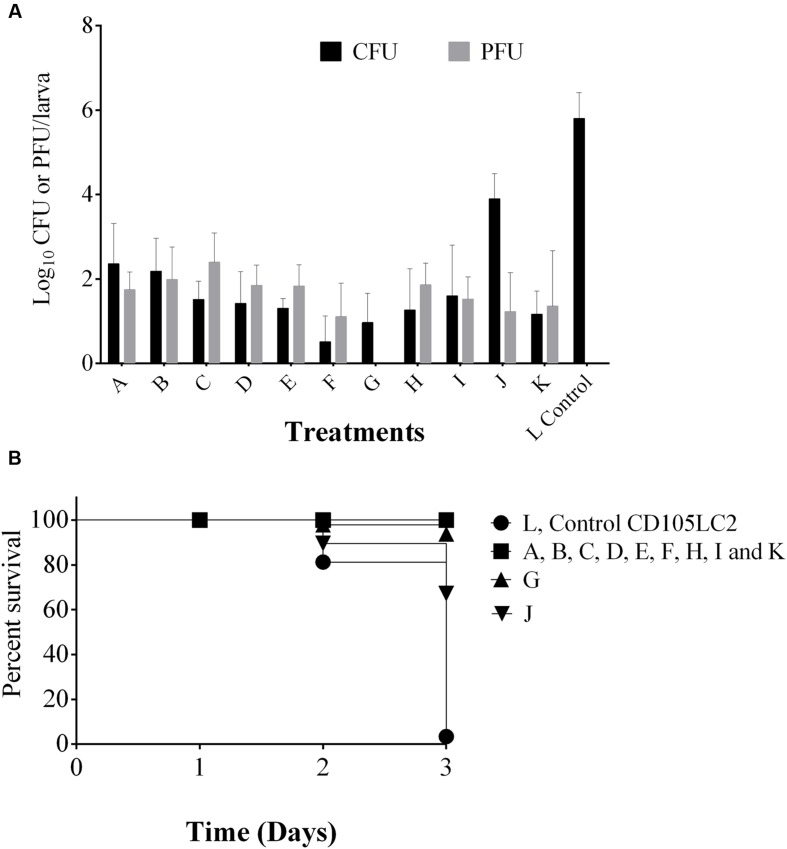
**Analysis of phage prophylactic regimens and impact of combined phage/vancomycin treatment on the *G. mellonella* larvae. (A)** Shows phage and bacterial recovery after prophylactic treatment with phages and delays of 2, 6, 12, and 24 h in groups A, B, C and D respectively were observed before bacterial exposure. Group E received phage followed by bacteria and vancomycin at the 2nd and 4th hour respectively. Group F was a reverse of group E. Group G, is vancomycin remedial treatment. H group represents combined phage/vancomycin prophylaxis and I group is combined phage/vancomycin remedial regimen. J and K regimen mimic dysbiosis where clindamycin was given first followed by bacteria then phage in J, but phages were given before bacteria in K. Larvae in bacterial control group L received only bacteria. **(B)** Represents survival rates in the treatments. Four larvae were examined in each group. Experiment was repeated three times. Error bars represent SEM of all replicates. Data was analyzed using GraphPad Prism 6.

We showed that the phages remain viable within the *Galleria* heamolymph for up to 5 days. In experiments shown in **Figure 5**, phages were administered 2 h prior to bacterial infection. Here, to determine the impact of giving phages to insects for longer times before bacterial inoculation prophylactic periods of 2 h (A), 6 h (B), 12 h (C), and 24 h (D) prior to bacterial infection were examined. Interestingly, increased phage activity resulted in increased phage counts in the 2–12 h delay times and corresponding decreased bacterial counts as the time delay prior to bacterial infection increased (~10^2^ CFU/larva at 24 h delay as opposed to ~10^3^ CFU/larva at 2 h delay) (**Figure 7A**).

When the phages were tested as adjuncts to vancomycin, but given prior to bacterial challenge, vancomycin was less effective at removing *C. difficile* (with ~10^2^ CFU/larva cells recovered) (E) than if phages were not given. In contrast, when vancomycin was given prophylactically before infection, the phages were more effective, leading to only ~10 CFU/larva recovered and this therapy (F) is more effective than vancomycin treatment (G), vancomycin/phage prophylaxis (H) and remedial treatment (I) in which ~10^2^ CFU/larva were recovered in both regimens (**Figure 7A**).

To mimic dysbiosis, clindamycin was given to the insects before bacterial infection followed by phage treatment (J). Here, we saw a proliferation of bacterial growth with ~10^5^ CFU/ml of bacteria being recovered. However, if phages were given post-clindamycin treatment and before bacterial infection (K), only ~10^2^ CFU/larva of bacteria were detected, which is comparable to the combined phage/vancomycin prophylaxis (**Figure 7A**).

In terms of survival, larvae in groups A, B, C, D, E, F, H, I, and K all survived but ~10 and 30% of the larvae died in the vancomycin treatment and clindamycin pre-treatment 1 (G and J) respectively (**Figure 7B**).

## Discussion

*Clostridium difficile* is a notorious nosocomial pathogen causing fatalities in the immunocompromised. A limiting factor to the control of the disease is the lack of treatment options (Zucca et al., 2013; Hargreaves and Clokie, 2014). *C. difficile* produces spores and biofilms that significantly contribute to its virulence by enabling persistence and proliferation in the environment and gut, and resistance to antimicrobial and cleaning agents (Akerlund et al., 2008; Burns et al., 2011; Burns and Minton, 2011; Dawson et al., 2012; Đapa et al., 2013). Phages are antimicrobial agents that can penetrate biofilms and could potentially be developed to supplement the currently available CDI treatments for better efficacy and clinical outcome (Cos et al., 2010; Morten et al., 2011). Here, we examined the impact of an optimized phage combination on *C. difficile* biofilms and tested their efficacy both as a stand-alone treatment and as adjunct to vancomycin in a *G. mellonella* larva CDI model. We showed that the phages can penetrate well-established *C. difficile* biofilms leading to lysis, plaque formation and a reduction in bacterial viability and biomass *in vitro*. A single phage treatment is effective in the pre-biofilm treatment *in vitro*, and prophylactically in the insect models, although multiple phage doses were needed to improve the remedial regimen.

### *C. difficile* Phages Could Target Biofilms of a Prevalent Ribotype 014/020 Strain

To evaluate the potential efficacy of our phages for CDI treatment, we targeted a clinically prevalent and severe ribotype strain (CD105LC2, ribotype 014/020) (Indra et al., 2015; Lessa et al., 2015). In addition to the pathogenicity of CD105LC2, our previous data showed that the individual phages in the mix have optimal plaquing efficiencies on the strain and this combination could completely eradicate *C. difficile in vitro* and reduce its colonization in a CDI hamster model (Nale et al., 2016). Having previously established the efficacy of the phages on *C. difficile in vitro* and on a hamster model, here we went further to test their effect on the biofilm and insect models. This is to determine if they are useful models in which to probe different aspects of *C. difficile*-phage interaction which can inform their future development, and of course useful models for the development of other phages that target biofilm-producing bacteria.

### Optimized Phage Combination Is More Effective at Removing *C. difficile* Biofilms Compared to Single Phage Treatments

When testing the efficacy of the phages on biofilms we showed that the phage combination is more efficient at reducing biofilms compared to using the single phages. This observation concurs with previous findings, which showed that treatment of *C. difficile* with single phage is commonly associated with the emergence and proliferation of resistant or lysogenic clones (Ramesh et al., 1999; Meader et al., 2010, 2013; Govind et al., 2011; Nale et al., 2016). Like all previously characterized *C. difficile* phages, the phages examined in this study have integrases in their genomes and could potentially access the lysogenic pathway (Goh et al., 2013; Hargreaves and Clokie, 2014). However, our previous data using the optimized phage combination *in vitro* suggest that the effect of resistance or lysogeny could be circumvented through complementation (Nale et al., 2016). The data presented here further supports this observation as the combined phages caused clear patches of killing on the biofilms.

### *C. difficile* Phages Are Effective Pre-biofilm Agents

We investigated three different pre-biofilm treatments with the combined phage mixture. In all cases, a significant biofilm reduction was observed. The clearance could be attributed to the ability of the phages to either completely eliminate or reduce the bacteria even before the biofilms were formed (Chan and Abedon, 2015; Nale et al., 2016). When the phages were incubated at 37°C for an hour before the bacterial cultures were added (Set 3), bacteria were eliminated beyond limit of detection. Similar observation was made when catheters were pre-treated with phages before exposure to *Pseudomonas aeruginosa* biofilm (Fu et al., 2010). This could be attributed to the phages being able to acclimatize to the incubation temperature and conditioned to optimum bacteriolytc activity before the bacterial culture was added (Taj et al., 2014). Temperature plays key roles in the attachment, adsorption, latent period, and multiplication of phages, and thus could greatly affect their infectivity (Olson et al., 2004; Jończyk et al., 2011). Efficacy was not as significant in Sets 2 and 4, where the phages were added directly from storage (at 4°C) to the culture at 37°C. Although the phages maintain a stable latent period in storage, at lower than optimal bacterial growth temperature, reduced attachment may take place leading to decreased phage activity (Jończyk et al., 2011). The temperature change encountered by the phages after being transferred to 37°C could have retarded their initial activity, allowing the bacteria to multiply and thus led to reduced MOI, and consequently recovery of bacteria in the phage-treated culture. The reduced effect on the established biofilm could potentially be attributed to less preference of phages to mature biofilms (Abedon, 2016) or the negative impact of PBS used for the biofilm washings on the activity of the phages (Silva et al., 2014). Our preliminary data showed that the stability of the phages in PBS is greatly reduced by ~ 2 logs CFU/ml by 1 h (Supplementary Figure S5). This observation is in agreement with a previous report (Silva et al., 2014).

### Phages Could Impact Established *C. difficile* Biofilms

Although we observed that the phages impacted the established biofilms less than the pre-treated cultures, confocal and scanning electron microscopy data both showed that the phages did penetrate the – biofilms and clear phage plaques were formed (**Figure 2**) (Abedon, 2016). In addition to the microscopy observations, further evidence to suggest that the phages penetrate the biofilm was indicated by the reduction in the vegetative cell counts on up to day-4 biofilms. Sometimes, phage lysis of biofilms is mediated by certain phage proteins which could chemically degrade the extracellular polymeric substance (EPS) found in the biofilm (Chan and Abedon, 2015). Previous work conducted on *C. difficile* phages has shown that purified endolysin is effective at clearing of the bacteria. Evidence to suggest its activity on *C. difficile* biofilms is yet to be confirmed (Mayer et al., 2008; Mayer and Narbad, 2012). Data from our SEM analysis revealed that the clearance was restricted to healthy vegetative cells only, thus we could attribute the clearance to lysis from phage infection (Chan and Abedon, 2015; Abedon, 2016).

The observation of elongated dead cells in the lysed zones is consistent with other findings that associated bacterial elongation or plasticity with loss of normal cell division process, changes in metabolic processes or DNA damage (Justice et al., 2008; Ghaffar et al., 2015). This morphological change is associated with stress conditions. Thus, conditions such as antimicrobial treatments or starvation and have been reported in bacteria such as *Helicobacter pylori*, *Campylobacter* and *Listeria monocytogenes* (Takeuchi et al., 2006; van der Veen et al., 2010; Ghaffar et al., 2015) and could trigger cell lysis (Kohanski et al., 2010). In *C. difficile*, cell elongation was reported to be associated with stress from treatment of the cells with sub-MIC of ridinilazole, which caused the cells to increase from 4 μm to 100 μm in length after 24 h treatment with the antibiotic (Bassères et al., 2016). Although the mechanism of ridinilazole is not clear, this response has been attributed to the SOS response and cell death from activity of β-lactam antibiotics (Kohanski et al., 2010). This bacterial response to antimicrobial stress, leading to cell elongation and death could possibly explain our observation of filamentous cells at 24 h.

### Phages Are Effective Prophylactic Therapeutics and Adjuncts to Vancomycin in *G. mellonella* CDI Model

We investigated the efficacy of the combined phages alone and as adjuncts to vancomycin in *G. mellonella* larvae. Although the insect is used as an infection model for various pathogens (Mukherjee et al., 2010; Ramarao et al., 2012; Hill et al., 2014; Beeton et al., 2015) and antimicrobial testing (Seed and Dennis, 2009; Abbasifar et al., 2014; Beeton et al., 2015; Olszak et al., 2015), to our knowledge this report is the first published data to describe its use to study *C. difficile* colonization and phage treatment. We established that 10^5^ *C. difficile* cells were sufficient for colonization to occur in the larvae and to cause the first and total mortality at 24 and 60 h respectively post-infection. The choice of the inoculum dose and timing was optimal to allow phage/vancomycin therapies to take place. Also, the bacterial inocula we used were suspended in BHI broth. Previous reports showed that bacteria were suspended either in PBS (Ramarao et al., 2012; Hill et al., 2014) or in 0.9% NaCl (Mukherjee et al., 2010, 2013). However, we could not use either of these solutions since our phages are unstable in PBS, and the NaCl solution negatively interfered with bacterial growth. Therefore, the bacteria were re-suspended in cold BHI broth, which is the growth medium for the bacteria and in which the phages are stable. The BHI broth control is comparable to PBS and NaCl as shown by the larval survival data (Supplementary Figure S2).

We conducted our phage treatment at MOI of 10 to allow comparison of the data from the current study with our previous report (Nale et al., 2016). Work is ongoing to examine other MOIs. Although previous work reported the inoculation of bacteria/phages by intrahaemocoelic injection (Abbasifar et al., 2014; Beeton et al., 2015), we found ingestion via the oral route more efficient at inducing colonization and producing reproducible therapy results. The observed higher efficacy of the phages when used prophylactically rather than with remedial treatment is consistent with a previous report (Beeton et al., 2015). Interestingly, the best treatment was observed when vancomycin was given prophylactically followed by phage application. This is significant as it could potentially serve as an intervention therapy to supplement vancomycin and prevent disease relapse. Similarly, the effectiveness of increased window time of having phages prior to bacterial infection could also be attributed to the ability of the phages to be distributed throughout the gut and acclimatize to the incubation temperature (Jończyk et al., 2011; Beeton et al., 2015) for effective bacteriolysis. The increased survival rates and corresponding decreased bacterial counts observed with the multiple phage dose regimens could be attributed to increased number of phages consequently leading to increased MOI and activity.

## Conclusion and Recommendations

The optimized phage combination studied here showed great potential in the control of *C. difficile* biofilms. And the biofilm model is useful for the development of phages that target both this and other pathogenic bacteria. Pre-treating likely surfaces, or potentially, cell lines is most effective as no colonization occurred after their application. Again in the insect model, the phages demonstrated a clear prophylactic potential as a stand-alone treatment for CDI and as adjuncts to vancomycin in the remedial treatment. These characteristics of the phages could be explored further to prevent and control CDI.

## Author Contributions

JN, MC, and MRJC designed the experiments. JN, PC, and PH performed the biofilm assays. JN, MC, and PH performed the *in vivo* experiments. JN analyzed the data. JN, MC interpreted the results. JN drafted the manuscript. MC, PH, PC, and MRJC edited the manuscript. JN, MC, PC, PH, and MRJC agreed to be accountable for all aspect of the manuscript and approved the final version to be published.

## Conflict of Interest Statement

This work was funded by AmpliPhi Biosciences and under the terms of a license agreement the author Prof. MC, as an employee of the Universities of Leicester, may be entitled to a share of license revenue and therefore declare an interest. All the other authors declare that the research was conducted in the absence of any commercial or financial relationships that could be construed as a potential conflict of interest.
