# Supplementary material for: ‘Get in Early’; Biofilm and Wax Moth (Galleria mellonella) Models Reveal New Insights into the Therapeutic Potential of Clostridium difficile Bacteriophages
Source: Front Microbiol. 2016 Aug 31;7:1383. doi: 10.3389/fmicb.2016.01383 (PMC5005339; doi:10.3389/fmicb.2016.01383)
Supplement: Supplementary file 1 [file Data_Sheet_1.PDF]

Supplementary Data

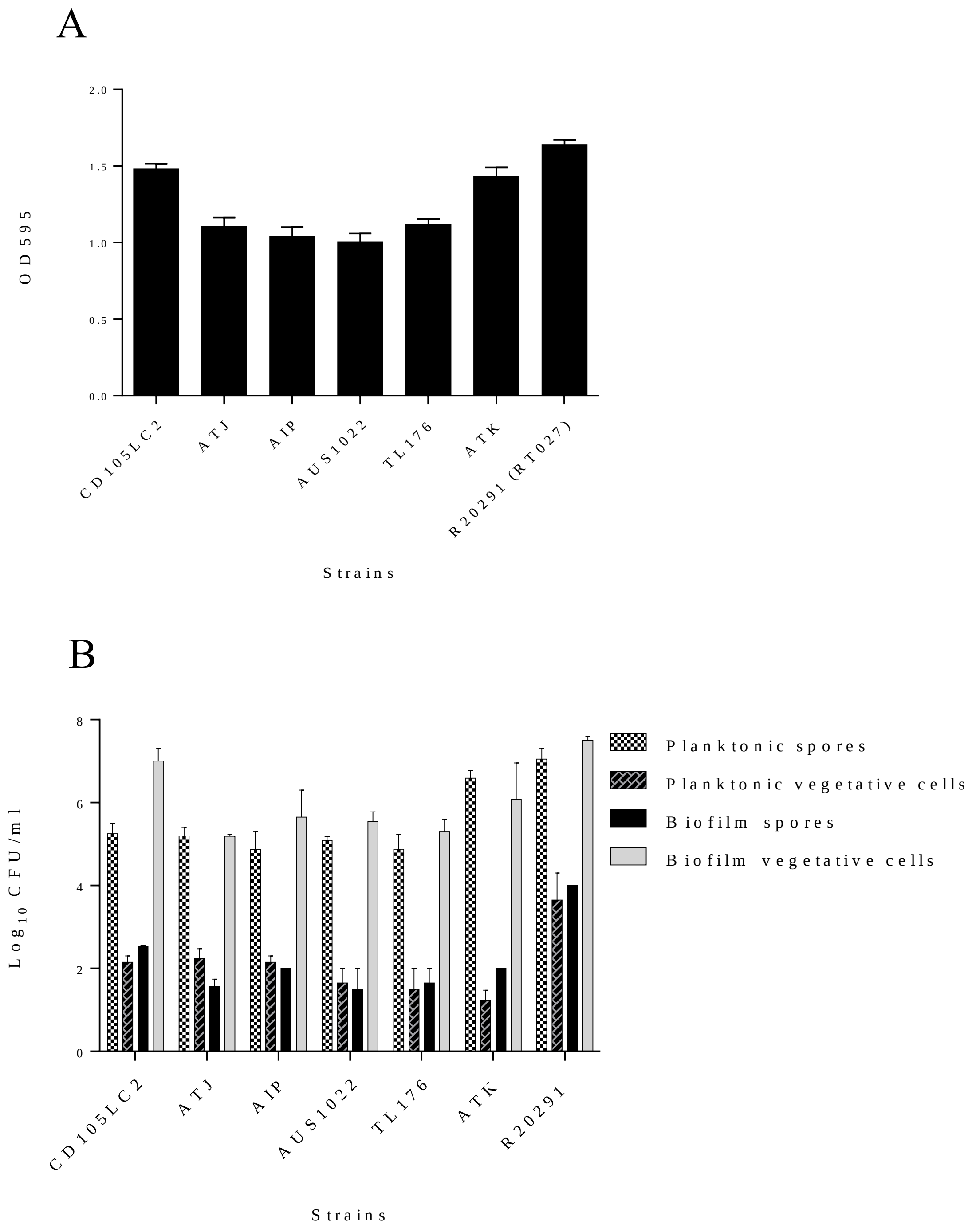

Figure S 1 Biofilm characteristics of *Clostridium difficile* strains.

A, Preliminary 24 h biofilm readings of *C. difficile* strains after crystal violet staining. Data was read at OD_{595}. **B**. Resultant viability counts of spores and vegetative cells in the biofilms and planktonic cultures are shown. Mean data of three biological replicates are represented. Experiment was repeated twice. Error bars are standard error mean of all replicates.

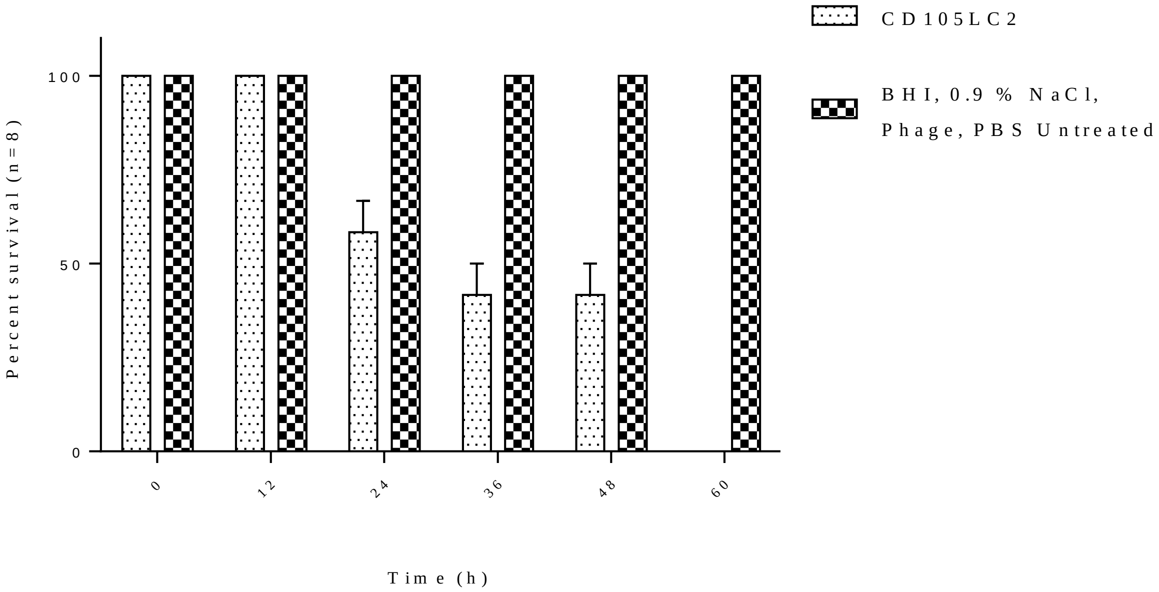

Figure S 2 Preliminary data showing survival rates of *G. mellonella* larvae treated with BHI, phages and NaCl and compared with bacterial (CD105LCS) and untreated control.

Larvae were inoculated with 10 µl of either BHI or 0.9 % NaCl, or BHI containing a total 10^5 CFU of bacteria or 10^6 PFU of phages. Insects were incubated at 37 ^{o}C and survival readings were taken 12 hourly for 60 h post-inoculation. Larva remained unfed throughout the duration of the experiment. Four larvae were examined in each group and the experiment was repeated twice. The error bars are standard error mean of all the replicates.

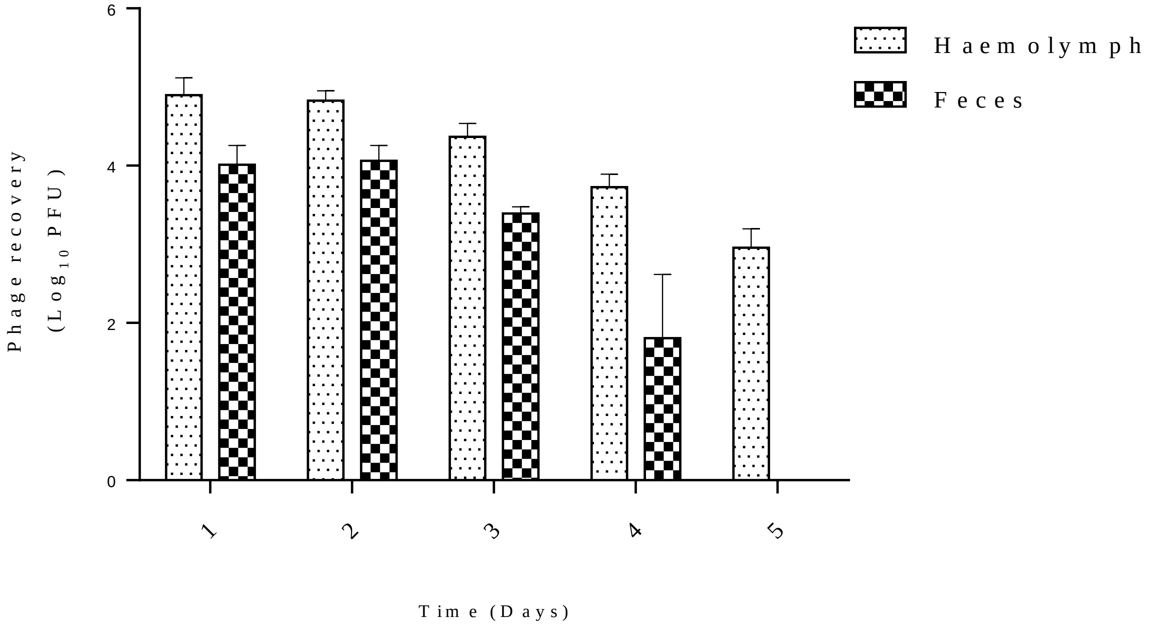

Figure S 3 Stability of the phages within *G. mellonella*.

Phages were inoculated via the oral route and the number of viable phages was ascertained from the larva and feces daily for up to 5 days. Phages were detected from the haemolymph of each larva while the fecal matter from 4 larvae in a Petri dish were combined together and assayed. To do this, the insects were first sedated by incubating them at -20 ^{o}C for 5 min before dissection dorso-ventrally. The gut extract of each larva were extracted into cold BHI and incubated for 1 h at 4 ^{o}C. After centrifuging at 15, 000 *g* for 5 min, the filtered supernatants were assayed for phages on a lawn of the propagating/indicator host, CD105HE1. Phage numbers per larva are represented. To extract phages from the feces, the fecal matter of 4 larvae in each Petri dish were combined and added into 1 ml of cold BHI. Phages were assayed as describe above. Phages from combined fecal matter of 4 larvae are presented. Experiment was repeated twice. Error bars represent standard error mean of all replicates.

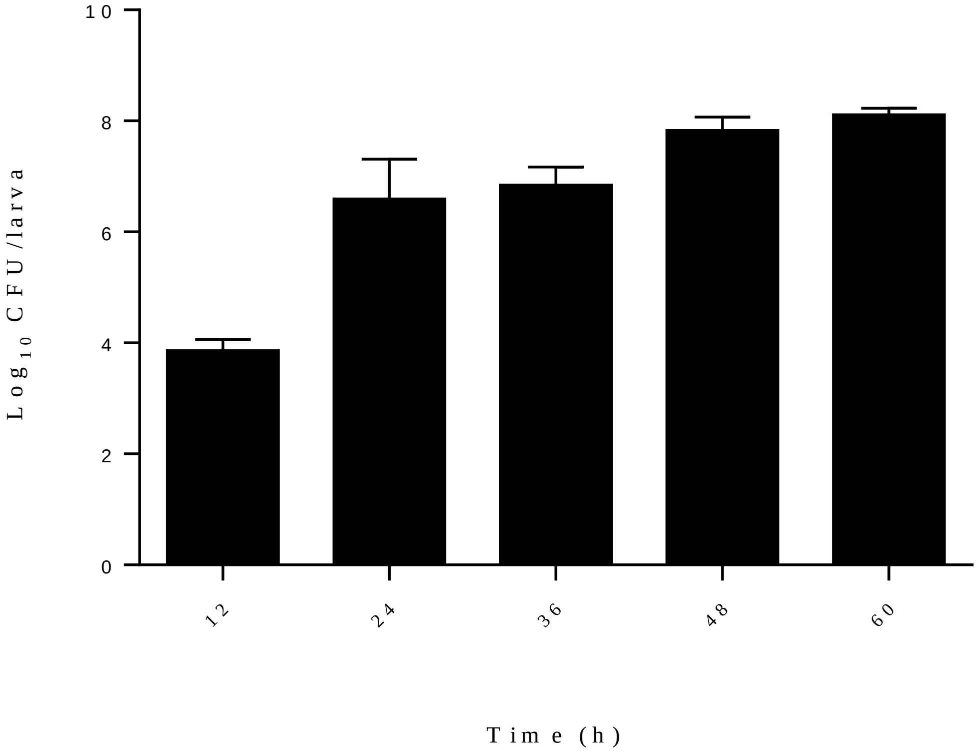

Figure S 4 Colonization of *C. difficile* CD105LC2 in *G. mellonella.*

A set of 4 larvae for each time point were inoculated with 10^5 CFU of bacteria via the oral cavity and incubated at 37 ^{o}C for 60 h. Larvae for each group were removed at the set time and the guts were extracted. The bacteria were enumerated by serial dilution in PBS and on CCEY plates. Experiment was repeated twice. Data for all the replicates are shown. Error bars are SEM of all replicates.

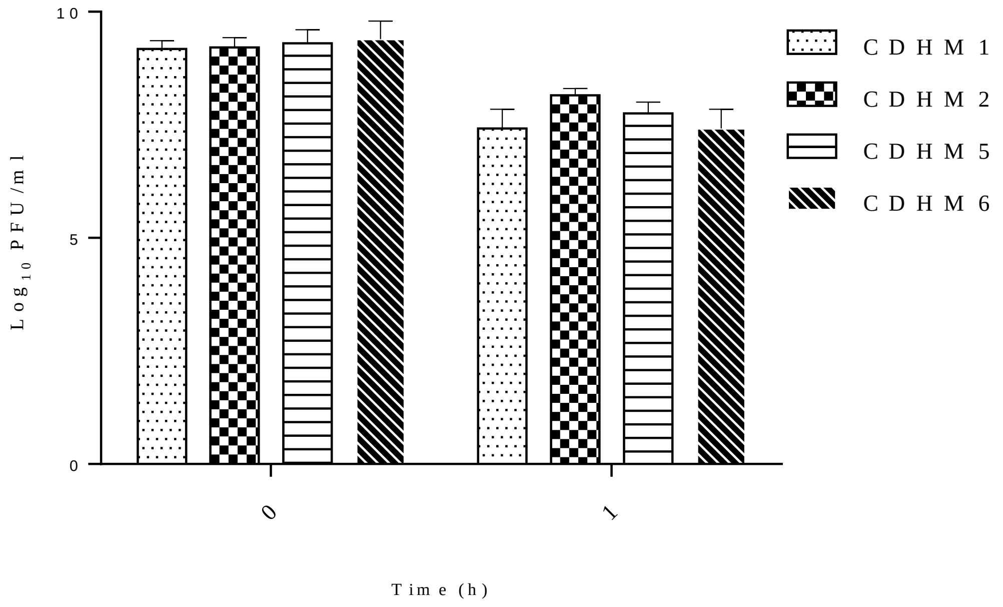

Figure S 5 Effect of phosphate-buffered saline (PBS) on the individual phages

Effect of PBS on the individual phages in the mixture was ascertained in PBS at 4 ^{o}C for 1 h. Viability counts before and after an hour incubation are shown. Samples were examined in triplicates and repeated twice. Bars are mean replicates of all the repeats.
